# Pyoderma gangrenosum in an abdominal surgical site: a case report

**DOI:** 10.1186/s40792-015-0124-3

**Published:** 2015-12-09

**Authors:** Kenichi Ogata, Hiroshi Takamori, Yoshiaki Ikuta, Hideyuki Tanaka, Nobuyuki Ozaki, Hiromitsu Hayashi, Katsuhiro Ogawa, Koichi Doi

**Affiliations:** Department of Surgery, Saiseikai Kumamoto Hospital, 5-3-1 Chikami, Munami-ku, Kumamoto 861-4193 Japan

**Keywords:** Pyoderma gangrenosum, Surgical site, Myelodysplastic syndrome

## Abstract

Pyoderma gangrenosum (PG) is an uncommon, ulcerative skin disease that is often associated with systemic diseases. Herein, we report a development of PG in a surgical site after cholecystectomy that was difficult to discriminate from surgical site infection. The patient was a 74-year-old man who had previously been diagnosed with myelodysplastic syndrome (MDS). Laparoscopic cholecystectomy was planned under diagnosis of cholecystolithiasis, but we converted to open cholecystectomy. The surgical wound was partially erythematous 4 days after surgery. In spite of opening the wound, cleansing it with sterile saline, and administration of antibiotics, inflammation spread with erosion. The clinical manifestations and histopathologic features of biopsy specimen indicated that diagnosis of PG associated with MDS was most likely. Administration of glucocorticoids made a rapid response of skin inflammation. The differential diagnosis of postoperative wound healing complications that were unresponsive to conventional wound local care and antibiotic therapy should include PG, especially in patients with systemic diseases such as MDS.

## Background

Pyoderma gangrenosum (PG) is an uncommon, ulcerative skin disease, and characterized by a rapidly enlarging necrotic ulceration with an undermined border and a surrounding of erythema [1–5]. It is often associated with systemic illness, such as inflammatory bowel disease (IBD), rheumatoid arthritis (RA), and myelodysplastic syndrome (MDS) [6, 7]. Recently, several manuscripts reported that PG could also occur after surgery and local trauma [8–10]. Herein, we report a development of PG in a surgical site after cholecystectomy that was difficult to discriminate from surgical site infection.

## Case presentation

**Fig. 1:**
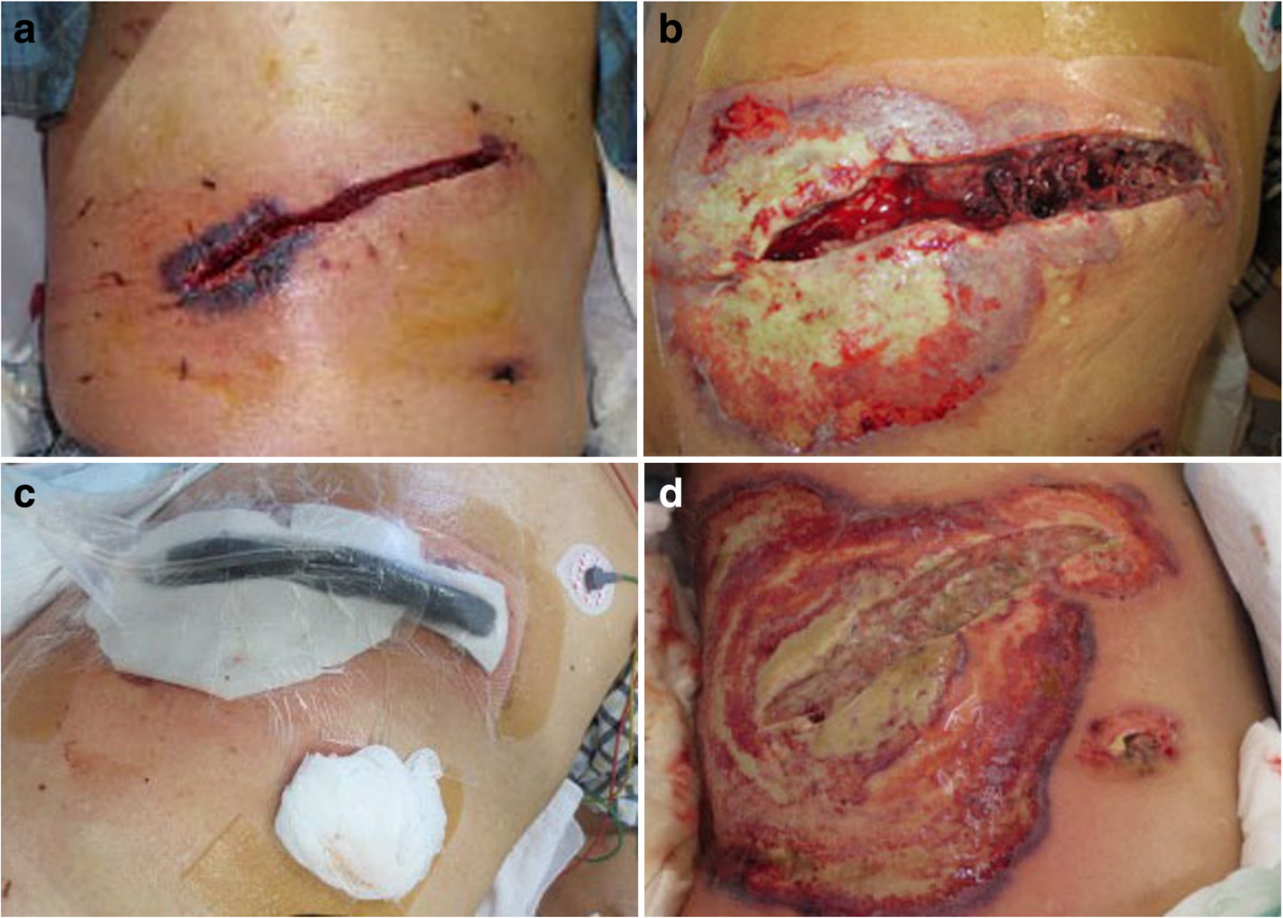
Erythema occurred in the surgical site 4 days after surgery (**a**). The skin inflammation change spread with erosion despite that moist environment dressing was introduced (**b**). The intra-wound continuous negative pressure and irrigation treatment (IW-CONPIT) was also introduced from 10 days after surgery (**c**). Inflammation continued to further spread with a purulent coating even after IW-CONPIT (**d**)

**Fig. 2:**
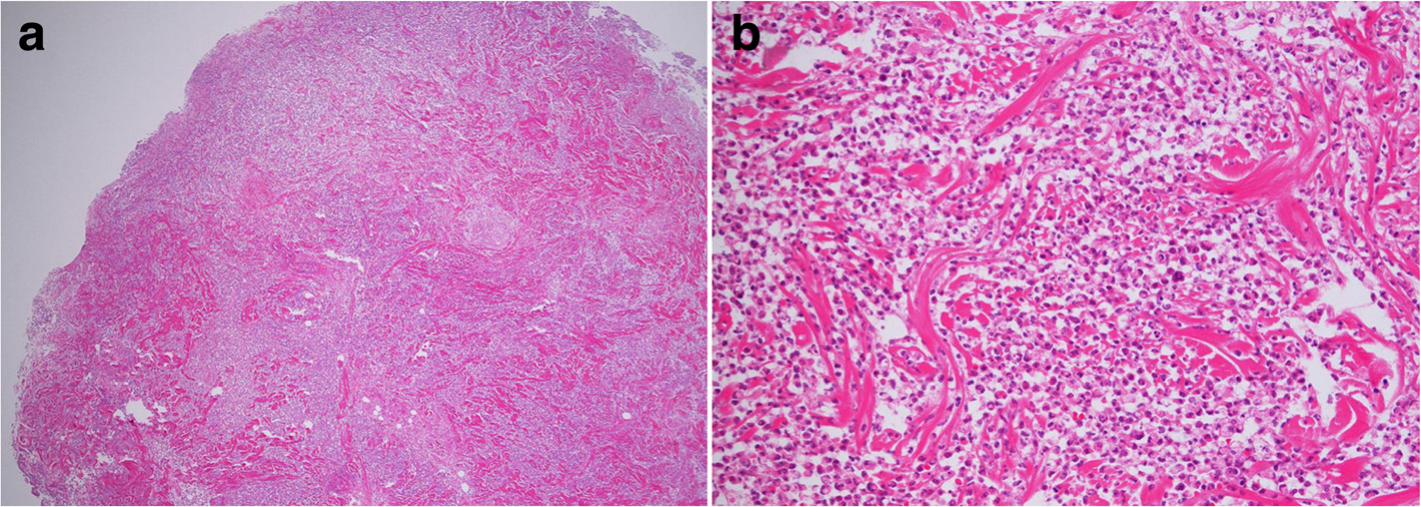
Histological findings of the skin biopsy revealed that severe inflammatory cell, predominantly of neutrophils, infiltrated in the dermis, and no bacterial components were observed. Hematoxylin-eosin stain ×40 (**a**), ×100 (**b**)

**Fig. 3:**
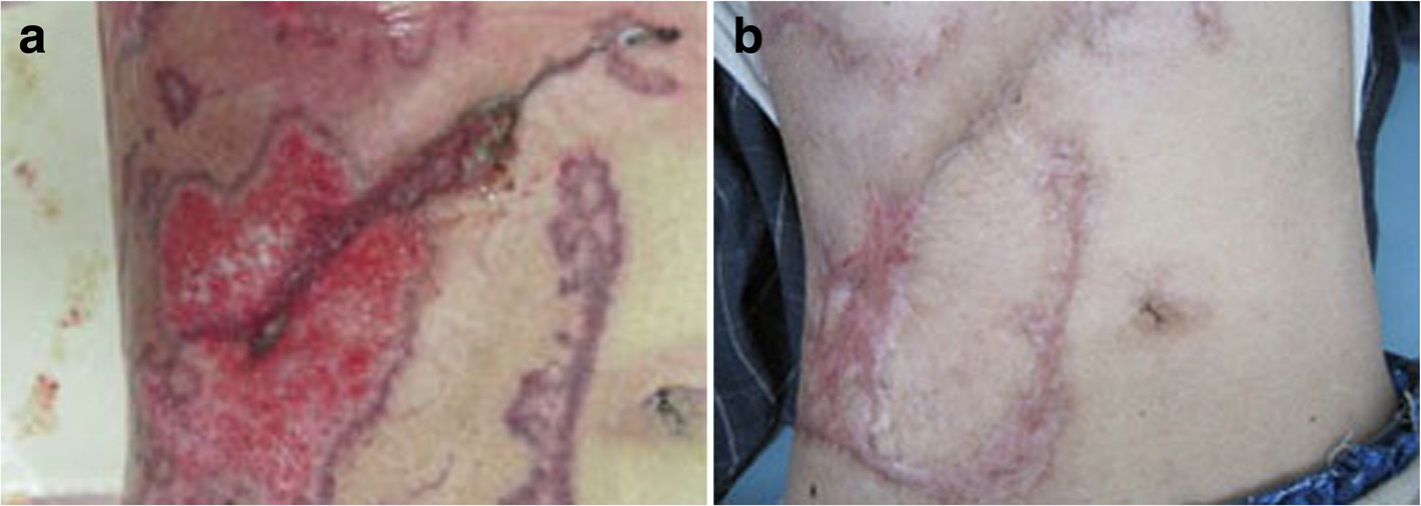
After the systemic administration of glucocorticoid, the purulent coating had disappeared on the eroded skin surface in the surroundings of the open wound. In addition, erosion had healed and there was promotion of epithelialization (**a**). One year after surgery, normal wound healing was achieved (**b**)

The patient was a 74-year-old man who had previously been diagnosed with MDS. He was diagnosed with calculous cholecystolithiasis at our hospital. Laparoscopic cholecystectomy was planned, but we converted to open cholecystectomy with a right subcostal oblique incision because it was difficult to remove the impacted calculus in the cystic duct. The surgical wound was partially erythematous 4 days after surgery (Fig. 1a), so we opened the wound and cleanse with sterile saline and performed moist environment dressing because of surgical site infection suspected. Inflammation, however, spread to the surrounding skin with erosion (Fig. 1b). The condition of wound rapidly deteriorated, so we introduced intra-wound continuous negative pressure and irrigation treatment (IW-CONPIT) 10 days after surgery (Fig. 1c). In spite of IW-CONPIT, inflammation continued to further spread widely with a purulent coating (Fig. 1d). In addition, skin edema spread from the lower back to the lower extremities. Antibiotic administration and irrigation were not effective, either. Bacteriological examination revealed that *Enterococcus faecalis* and *Pseudomonas aeruginosa* were detected in the wound, although those counts were low. We decided to perform biopsy of the skin surrounding the wound to analyze pathological condition. Histopathologic feature showed that severe inflammatory cell, predominantly of neutrophils, infiltrated in the dermis, and no bacterial components were observed (Fig. 2a, b). These clinical and histopathologic findings of the surgical site indicated that diagnosis of PG associated with MDS was most likely. After the addition of a systemic administration of 30 mg/day prednisolone to local care of the surgical site with moist dressing, fever had been rapidly alleviated, spread of rash had diminished, edema in the lower body had improved, and the purulent coating had disappeared on the eroded skin surface surrounding the open wound (Fig. 3a). Elevated CRP (18.1 mg/dL) and WBC counts (40.5 × 10^3^ μL) before administration of prednisolone had been improved remarkably, and these data became within normal ranges after 2 weeks, so the dose of prednisolone was tapered to 25 mg/day. The dose had been reduced 5 mg/day per 1–2 months to follow up the skin and general condition, including laboratory data. Erosion had healed and epithelialization occurred. The surface of the wound was covered with satisfactory granulation tissue, and normal wound healing was achieved after administration of 5 mg/day prednisolone for 1 year (Fig. 3b).

### Discussion

PG is an uncommon, chronic, recurrent, and painful cutaneous ulcerative disease with a distinctive morphologic presentation and uncertain etiology. The lower extremities are most common sites of involvement, though rare subtype of cases appears stoma site on ulcerative colitis increased recently [11]. More than half of patients with PG suffer from an associated systemic disease such as IBD, RA, and MDS [6, 7]. In this case, there was a history of MDS: it was to help diagnosis of PG.

Development of PG in a surgical and trauma site is rare and occurs mainly within 2 weeks after surgery [12]. The initial symptoms are surgical site erythema and extreme pain out of proportion to the physical examination [13–17]. PG is often initially diagnosed as a surgical site infection, though treatment with antibiotics and wound debridement fails to arrest rapid ulcer enlargement. Our case was also regarded as infection to treat with antibiotics, open drainage, and continuous wound lavage, but the condition had been getting worse. We performed excisional biopsy to clarify pathogenesis of the cutaneous lesion. Histopathologic findings revealed non-specific and neutrophil-dominant inflammation, but bacterial infection could be denied. The clinical and histopathological manifestations indicated that diagnosis of PG associated with MDS was most likely. Histological examination should be performed to exclude other disorders such as vasculitis, pyoderma, and vasculopathies, when the administration of antibiotic, debridement, and irrigation for inflammatory surgical site is ineffective and makes clinical exacerbation. A diagnosis of PG could be made when other diagnostic possibilities had been excluded, because no accepted diagnostic criteria existed. Su WP et al. proposed diagnostic criteria, including clinical and histopathologic findings and treatment response [18]. Post-surgical PG occurred after breast (25 %), cardiothoracic (14 %), abdominal (14 %), and obstetric (13 %) surgeries [19], could deny infection and other possibilities, suspected PG, and performed experimental administration of steroid, following respond dramatically. PG has a good prognosis if appropriate therapy is immediately selected for it, but unless it is done, its prognosis becomes worse rapidly; it is likely to progress to death with consequent sepsis [20].

Definitive guidelines for treatment of PG are lacking. Patients with PG are treated with local and/or systemic therapies. First, to make an optimal environment for wound healing, a moist wound environment after cleansing with sterile saline is required [21]. Local administration of corticosteroids could be used in patients with mild PG, though the efficacy of these drugs is limited to a few retrospective studies and case reports [22, 23]. In contrast, systemic therapy is necessary in patients with more severe PG. Glucocorticoids are mostly selected for systemic drugs [22, 24]. Zuo et al. reported that most patients were treated with oral prednisolone (0.5–1.5 mg/kg/day) or intravenous methylprednisolone (0.5–1 mg/kg/day) combined with/without immunosuppressants such as systemic or topical tacrolimus [19]. We administered oral prednisolone 30 mg/day (0.5 mg/kg/day) as initial treatment and by means of diagnostic administration. If this administration is not effective, the dose of oral prednisolone could increase or administrations of intravenous methylprednisolone and tacrolimus ointment could be selected. Topical tacrolimus is one of the effective local treatments for PG [25]. Cyclosporine exhibited equivalent effect compared with steroids as a systemic treatment in a randomized trial [26]. A wide variety of other systemic immunomodulatory drugs, including anti-tumor necrosis factor alpha agents could be also utilized as alternative or adjunctive treatments in patients with PG that fails to respond to glucocorticoids [2, 27].

## Conclusions

The differential diagnosis of postoperative wound healing complications that were unresponsive to conventional wound debridement or antibiotic therapy should include PG, especially in patients with systemic diseases such as MDS. Glucocorticoids make a rapid response in patients with postoperative PG.

## Consent

Written informed consent was obtained from the patient for publication of this case report and any accompanying images. A copy of the written consent is available for review by the Editor-in-Chief of this journal.

## Abbreviations

IBD: inflammatory bowel disease
IW-CONPIT: intra-wound continuous negative pressure and irrigation treatment
PG: pyoderma gangrenosum
MDS: myelodysplastic syndrome
RA: rheumatoid arthritis
